# Liquiritigenin Protects Rats from Carbon Tetrachloride Induced Hepatic Injury through PGC-1*α* Pathway

**DOI:** 10.1155/2015/649568

**Published:** 2015-06-25

**Authors:** Yiping Zhang, Yuanqiao He, Hongbo Yu, Fuying Ma, Jianguo Wu, Xiaoyu Zhang

**Affiliations:** ^1^Key Laboratory of Molecular Biophysics of Ministry of Education, College of Life Science and Technology, Huazhong University of Science and Technology, Wuhan, Hubei 430074, China; ^2^School of Basic Medical Science, Jiujiang University, Jiujiang, Jianxi 332000, China; ^3^Center of Laboratory Animal Science, Nanchang University, Nanchang, Jiangxi 30006, China

## Abstract

The lack of effective treatment for liver cirrhosis and hepatocellular carcinomas imposes serious challenges to the healthcare system. Here, we investigated the efficacy and mechanism of liquiritigenin involved in preventing or retarding the progression of liver diseases in a rat model with chronic carbon tetrachloride (CCl4) exposure. Sprague Dawley rats were given CCl4 and lliquiritigenin alone or simultaneously for 8 weeks before liver was harvested to check histological changes by Hematoxylin and Eosin (H&E) staining, apoptosis by TUNEL assay, ROS by dihydroethidium staining, antioxidant enzyme activities and malondialdehyde using specific kits, and gene expression by quantitative real-time PCR and western blot. Chronic CCl4 exposure caused profound changes in liver histology with extensive hepatocyte death (necrosis and apoptosis), fat accumulation, and infiltration of inflammatory cells, accompanied by depressed activities of antioxidant enzymes, increased oxidative stress, elevated expression of inflammation and fibrotic genes, and downregulation of PGC-1*α*, ND1, and Bcl-x in rat liver. All these changes were abolished or alleviated by lliquiritigenin. The results demonstrated that liquiritigenin is effective in protecting liver from injury or treating chronic liver diseases. The modulation of PGC-1*α* and its downstream genes might play a critical role in relieving CCl4-induced hepatic pathogenesis by liquiritigenin.

## 1. Introduction

Primary liver cancer with the majority of the cases being hepatocellular carcinoma (HCC) is one of the most common malignancies and has become the second leading cause of cancer death worldwide [1, 2]. The dominant risk factor of HCC is liver cirrhosis, which most frequently resulted from chronic hepatitis B virus (HBV) or hepatitis C virus (HCV) infection [2, 3]. Liver cirrhosis on its own is another significant public health issue, with up to 10% prevalence in general population and over 750000 annual deaths [4]. Surgical resection of early stage HCC and liver cirrhosis is superior over transarterial chemoembolization and becoming increasingly popular [5]. However, efficient noninvasive interventions are still sought, especially for later stage HCC and cirrhosis.

As the major organ to metabolize and detoxify metabolites and xenobiotics, the liver is liable to the damage caused by the hepatotoxicity of chemicals and oxidative stress [6]. Oxidative stress has been increasingly believed to play a crucial role in the pathogenesis of liver diseases [7]. While human and animal liver is capable of repairing damage with compensatory regeneration, it partially or completely loses its functions due to cirrhosis or cancer resulting from liver fibrosis as the injuries exceed its repairing capacity [8]. Carbon tetrachloride (CCl4) is widely used to establish rodent models of chronic liver injury as it is converted to highly reactive metabolites by the cytochrome P450 in liver [9], which in turn reduce antioxidant enzymes activity and eventually lead to membrane lipid peroxidation [10]. Therefore, reducing or eliminating reactive oxygen species (ROS) and free radicals would be an effective strategy for fighting hepatotoxicity. Peroxisome proliferator-activated receptor gamma, coactivator 1 alpha (PGC-1*α*) is a master regulator of energy metabolism and inflammation [11, 12]; it regulates a wide range of genes involved in mitochondria biogenesis and antioxidant.

Herbal medicine has been attracting increased interest in protecting and/or treating liver diseases [9, 13–15]. Liquiritigenin is a flavonone from* licorice*, the dried root of* Glycyrrhiza glabra*. It exhibits estrogenic [16], choleretic [17], anti-inflammatory [18], and antitumor [19] effects. This study investigates the effect of liquiritigenin on CCl4-induced chronic liver injuries and the biochemical pathways involved.

## 2. Materials and Methods

### 2.1. Animals

All animal protocols were conformed to the ethic requirements stated in the Declaration of Helsinki and approved by the institutional animal care and usage committee of Huazhong University of Science and Technology. Sprague Dawley (SD) rats were purchased from the laboratory animal center of Huazhong University of Science and Technology and acclimatized to the facility for a week before experiments. The rats were randomly divided into 4 groups (15 rats in each group) and given food and water* ad libitum*. The control rats fed on normal chow only; CCl4 group rats received 6 mg/kg CCl4 subcutaneously 3 times a week; liquiritigenin group (Liqtn) rats received 10 mg/kg liquiritigenin (≥98%, Cat number G02, Jiangxi Herbfine Hi-tech Co., Ltd., Nanchang, China) by gavages 3 times a week; and the CCl4/Liqtn group rats received 6 mg/kg CCl4 subcutaneously and 10 mg/kg liquiritigenin by gavages 3 times a week. After 8 weeks of treatment, rats were sacrificed and the livers were harvested and treated for downstream studies.

### 2.2. Pathology Observation

The pathological changes of livers were observed after Hematoxylin and Eosin (H&E) staining. The slides were deparaffinzed and hydrated, stained in alum haematoxylin for about 5 min, rinsed with running tap water, differentiated with 0.3% acid alcohol, rinsed with running tap water, stained with eosin for 2 min, and dehydrated, cleared, and mounted before being observed under a microscope by a pathologist.

### 2.3. Cell Apoptosis Assay

The apoptosis of hepatic cells in rat livers was analyzed with a TUNEL (terminal deoxynucleotidyl transferase dUTP nick end labeling) kit from Boyetime (C1098, Shanghai, China) according manufacturer's instruction. Briefly, sections were fixed with 4% paraformaldehyde 30–60 min, washed with PBS twice for 10 min, and incubated with 0.1% Triton X-100 in PBS on ice for 2 min and then with 0.3% H_2_O_2_ in methanol at room temperature for 20 min to quench endogenous peroxidase, which was followed by three washes with PBS. 50 *μ*L of Biotin labeling solution (including TdT enzyme and Biotin-dUTP) was added on top of the sections, which were incubated at 37°C for 60 min in a humidified chamber, washed once with PBS, incubated with 0.1–0.3 mL of labeling termination solution at room temperature for 10 min, and washed three times with PBS. The sections were then incubated with 50 *μ*L of Streptavidin-HRP working solution at room temperature for 30 min, washed with PBS for 3 times, incubated in 0.2–0.5 mL of DAB working solution at room temperature for 5–30 min, washed 3 times with PBS, and observed and photographed.

### 2.4. Quantitative Real-Time Polymerase Chain Reaction (qPCR)

Total RNA was isolated from rat liver using Trizol reagent (Invitrogen, Shanghai, China) according supplier's protocol. The reverse transcription was done with 1 *μ*g of total RNA using PrimeScript 1st strand cDNA Synthesis Kit (Takara, Beijing, China). Quantitative real-time PCR was performed using SuperReal PreMix (SYBR Green) (FP204, Tiangen Biotech, Beijing, China) on ABI7300 (Applied Biosystems, Foster City, CA). The primers were AAGGATGGAGGCACGATTGG and GGGAACTTGATGATGGGCGA for MMP-2; TCGCCACCGGATTGAAGAAA and CTCGGGAAGGCACAGCAATA for RelA; CGGTTTCCCGTGCAATCAGT and ACACCGGGGACCAAATGATG for GSH-Px; GGAGCAAGGTCGCTTACAGA and TCCCACACATCAATCCCCAG for SOD2; AGTCCCATACACAACCGCAG and CCCTTGGGGTCATTTGGTGA for PGC-1*α*; AATTCCTCTGGCCTGCCTAC and ATCATAGGTCGGGAGGAGGT for Bcl-x; ATGGCCTTCCTCACCCTAGT and GGGTTGGGGCGATAATAAAT for NADH dehydrogenase 1 (ND-1); and AACTCCCATTCCTCCACCTT and GAGGGCCTCTCTCTTGCTCT for GAPDH. The cycling program was 95°C 3 min followed by 40 cycles of 95°C 30 sec, 58°C 15 sec, and 68°C 30 sec. The relative mRNA levels were calculated using 2^−ΔΔCt^ method with GAPDH as the internal control.

### 2.5. Western Blot

Rat liver proteins (40 *μ*g) were resolved on 8% SDS-polyacrylamide gels and transferred onto PVDF membranes, which were blocked with 5% non-fat milk in PBST (0.5% Triton X-100 in PBS) for 30 min at room temperature and incubated with specified antibodies overnight at 4°C. The membranes were washed with PBST, incubated with horseradish peroxidase conjugated secondary antibodies at room temperature for 60 min, washed, and visualized with ECL reagent. First antibodies were RelA (112A1021) from Novus Biologicals (Littleton, CO), MMP2 (AF1488) from R&D Systems (Shanghai, China), PGC-1*α* (SAB4200209) from Sigma-Aldrich (St. Louis, MO), Bcl-x (ab32370) from Abcam (Cambridge, MA), and *β*-Actin (20536-1-AP) from Proteintech (Wuhan, China).

### 2.6. ROS Assayed with Dihydroethidium (DHE)

Hepatocytes were incubated with medium containing 10 mM DHE at 37°C in the dark for 15 min. The cells were washed twice with PBS before being observed under fluorescent microscope or analyzed by fluorescent activated cell sorting with band-pass filter at 585 nm. The mean fluorescence intensity was obtained from 10,000 cells.

### 2.7. Lipid Peroxidation Assay

The level of liver malondialdehyde (MDA) was measured using a kit (K739-100) from BioVision (Milpitas, CA) according to manufacturer's protocol. Briefly, 10 mg of liver sample was homogenized in 300 *μ*L of MDA Lysis Buffer (with 3 *μ*L 100X BHT) and centrifuged 10 min at 13000 g at 4°C. 200 *μ*L of the supernatant was added to an Eppendorf tube. MDA standard curve was set according to instruction to make 0, 4, 8, 12, 16, and 20 nmol standard. 600 *μ*L of TBA reagent was added to each tube containing sample or standard, incubated at 95°C for 60 min, and cooled down on ice for 10 min. 200 *μ*L reaction mixture from each tube was transferred into a 96-well microplate and read at a wavelength of 532 nm on a microplate reader (BioTek, Beijing, China).

### 2.8. Activities of Superoxide Dismutase Activity (SOD) and Glutathione Peroxidase (GSH-Px)

The activities of SOD (ab65354) and GSH-Px (ab102530) were analyzed using assay kits from Abcam (Cambridge, MA) according to manufacturer's instructions.

For GSH-Px activity measurement, liver tissues (10 mg) were washed with cold PBS and homogenized in 200 *μ*L cold Assay Buffer with a Dounce homogenizer on ice for about 15 passes and centrifuged at 10000 g for 15 min at 4°C and the supernatant was collected into a fresh tube. Assay plates were set up with standards, proper controls, and samples. 40 *μ*L of freshly made reaction mix was added to samples and controls and mixed and incubated at room temperature for 25 min. Then 10 *μ*L cumene hydroperoxide solution was added to each well, mixed, and read at 340 nm on a plate reader (BioTek). The plates were incubated in the dark at 25°C for 5 min and read again at 340 nm.

For SOD assay, 10 mg of liver tissue was washed in cold PBS to remove red blood cells and homogenized in cold 0.1 M Tris/HCl, pH 7.4 (containing 0.5% Triton X-100, 5 mM *β*-ME, and 0.1 mg/mL PMSF) and centrifuged for 5 min at 4°C at 14000 g, and then supernatant was transferred into a clean tube. The standards, controls, and samples were added to 96-well assay plates; then, 200 *μ*L of WST working solution was added to all wells and 20 *μ*L of Enzyme Working Solution was added to wells containing samples and proper controls only according to manufacturer's instruction. The plates were mixed and incubated 20 min at 37°C before being read on a microplate reader (BioTeK) at 450 nm.

### 2.9. Statistical Analysis

The data was presented as mean ± standard deviation. The differences between treatment groups and control were analyzed with one-way analysis of variance. It was considered statistically significant if a *P* value was less than 0.05.

## 3. Results

### 3.1. Liquiritigenin Alleviated CCl4-Induced Hepatic Injuries

**Figure 1:**
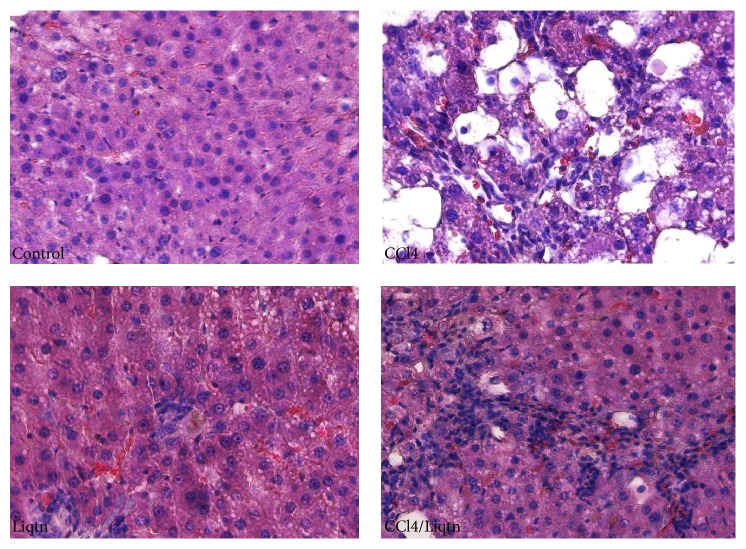
Liquiritigenin alleviated CCl4 caused the histological destruction of rat livers. Rats were treated with CCl4 and/or liquiritigenin for 8 weeks and liver sections were stained with Hematoxylin and Eosin. CCl4 treated rats had severe liver histological abnormity with widespread hepatocyte death, fatty accumulation, and immune cell infiltration, which was largely relieved by liquiritigenin.

**Figure 2:**
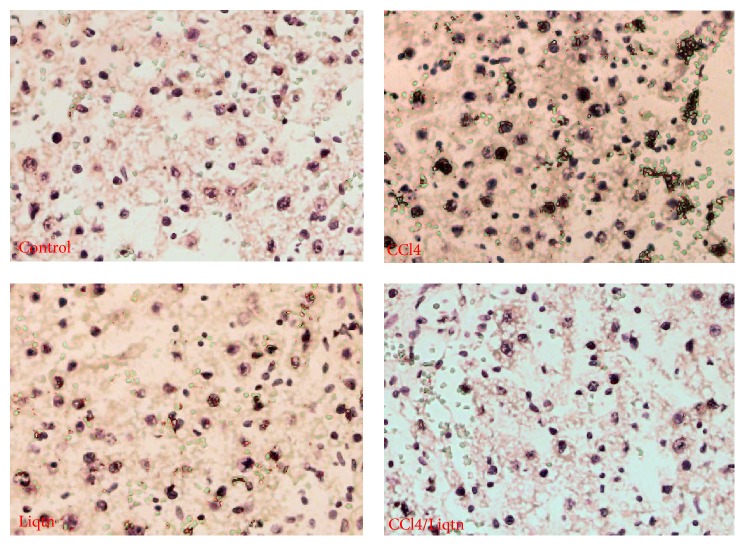
Liquiritigenin inhibited hepatocyte apoptosis in CCl4 treated rat livers. The apoptotic cells in rat liver sections were detected with a commercial TUNEL kit. The results showed increased apoptosis in rat liver exposed to CCl4 while liquiritigenin protected the liver cells from CCl4-induced apoptosis.

CCl4 treatment caused the loss of regular liver structure seen in control rat with widespread necrosis of hepatocytes, fatty accumulation, and significant lymphocytes infiltration. Liquiritigenin treatment significantly reduced the severity of CCl4-induced hepatic damages with much less necrosis of hepatocytes and few diffused fatty changes (Figure 1). Meanwhile, the number of apoptotic hepatocytes was markedly increased by CCl4 treatment, which was suppressed by liquiritigenin (Figure 2).

### 3.2. Liquiritigenin Relieved CCl4 Caused Oxidative Stress

**Figure 3:**
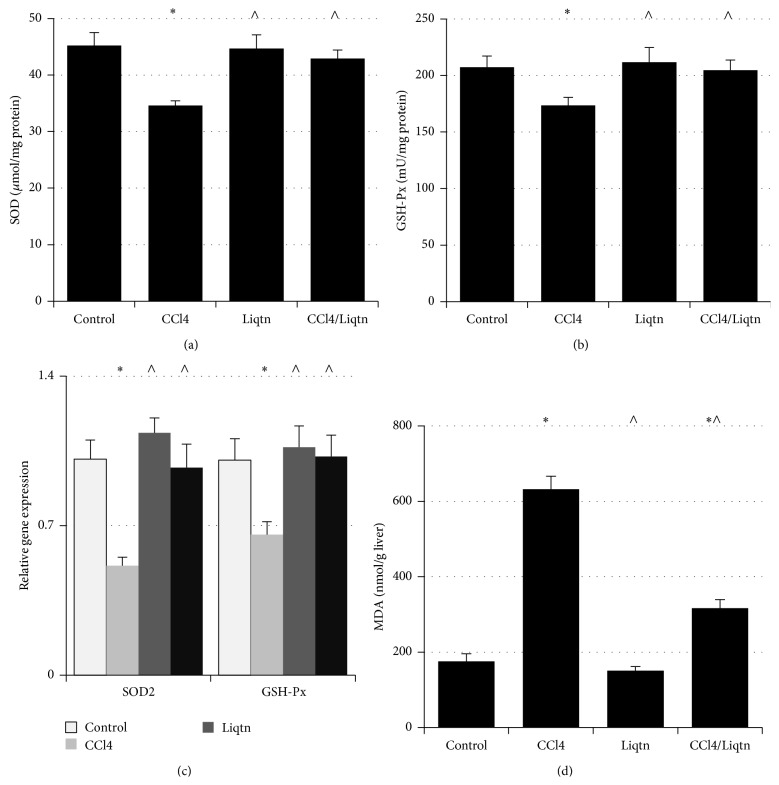
CCl4-illicited oxidative stress was effectively inhibited by liquiritigenin. The enzymatic activities of liver SOD (a) and GSH-Px (b) were analyzed with commercially available assay kits. (c) The mRNA levels of SOD2 and GPx in livers of CCl4 and/or liquiritigenin treated rats were assessed by quantitative real-time PCR. (d) The MDA (lipid peroxidation) level was analyzed using an assay kit. ^*∗*^ *P* < 0.05 compared to control rats; ^∧^ *P* < 0.05 compared to CCl4 treated rats.

Chronic CCl4 exposure significantly decreased the activities of superoxide dismutase (Figure 3(a)) and glutathione peroxidase (Figure 3(b)) as well as their mRNA levels (Figure 3(c)) in rat liver. The SOD and GSH-Px activities in the livers of rats exposed to chronic CCl4 were reduced by 23.5% and 16.3% compared to control rats, respectively (Figures 3(a) and 3(b)). Liquiritigenin treatment abolished CCl4-induced reduction of SOD (Figure 3(a)) and GSH-Px (Figure 3(b)) activities and their expression levels (Figure 3(c)). The SOD activity of rat livers treated with both liquiritigenin and CCl4 recovered from 34.6 *μ*mol/mg protein in CCl4-treated rats to 42.9 *μ*mol/mg protein (*P* < 0.05), which was similar to that of control rats (45.2 *μ*mol/mg protein) (Figure 3(a)). The activity of GSH-Px of liquiritigenin/CCl4 rat livers was 17.9% higher than that of CCl4 rat livers (*P* < 0.05, Figure 3(b)). The liver mRNA levels of SOD2 and GSH-Px of CCl4 treated rats were 57.3% and 65.8% of those of the control, which were improved to 97.1% and 102.3% of the control in rats that received liquiritigenin while being exposed to CCl4 (Figure 3(c)).

The lipid peroxidation levels were analyzed by measuring hepatic malondialdehyde levels in rats treated with CCl4 and/or liquiritigenin. Chronic CCl4 exposure caused an about 3.5-fold increase of MDA in rat livers (Figure 3(d)). Liquiritigenin treatment lowered liver MDA levels by almost 50% in CCl4-exposed rats (Figure 3(d)).

**Figure 4:**
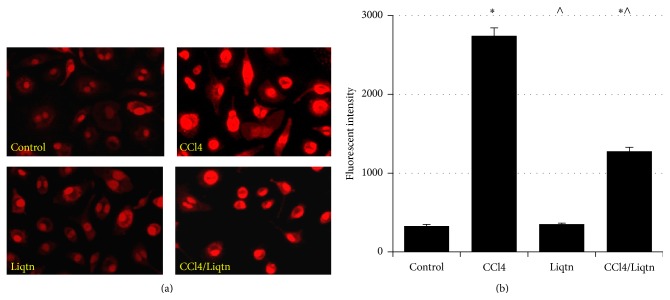
Liquiritigenin mitigated CCl4-induced ROS. The cellular ROS in rat livers treated with CCl4 and/or liquiritigenin was stained with dihydroethidium followed by fluorescence microscopy or flow cytometry. ^*∗*^ *P* < 0.05 compared to control rats; ^∧^ *P* < 0.05 compared to CCl4 treated rats.

Meantime, the reactive oxygen species level of hepatocytes from CCl4 treated rats was more than 8-fold higher than that of control rat hepatocytes. Liquiritigenin alone did not have effects on the ROS level of rat hepatocytes, but it reduced ROS levels by more than 53% compared to hepatocytes from rats exposed to CCl4 only (Figure 4).

### 3.3. Liquiritigenin Reduced CCl4-Induced Hepatic Inflammatory and Fibrotic Responses

**Figure 5:**
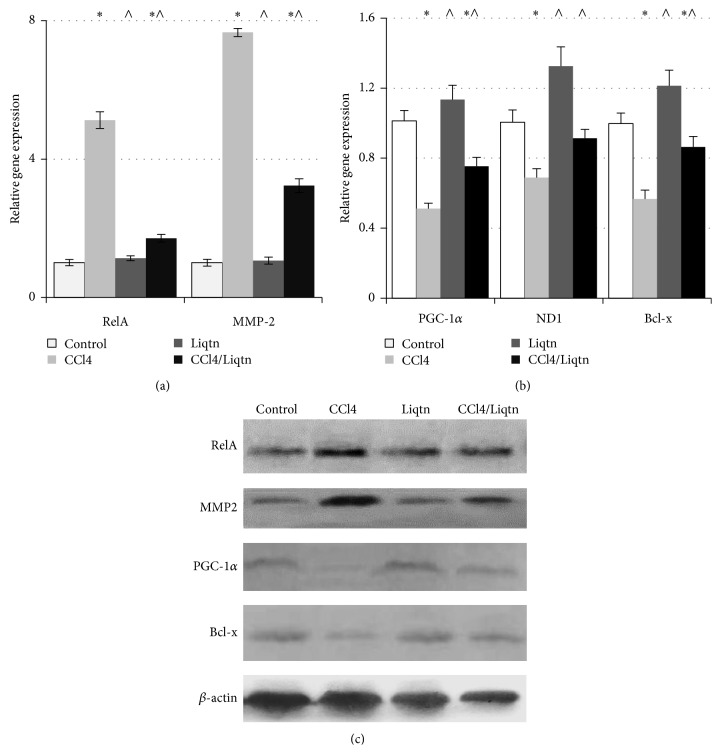
Liquiritigenin overturned the abnormal gene expression caused by CCl4. The expression of examined genes was analyzed by quantitative real-time PCR ((a) and (b)) and western blot (c). ^*∗*^ *P* < 0.05 compared to control rats; ^∧^ *P* < 0.05 compared to CCl4 treated rats.

The mRNA levels of RelA and MMP2 were increased by more than 5- and 7.5-fold, respectively, over control rats by CCl4 treatment, which was reduced by about 66% and 58% by liquiritigenin (Figure 5(a)). Consequently, the protein levels of RelA and MMP2 were significantly increased in livers of CCl4 treated rats and the increase was attenuated by liquiritigenin (Figure 5(c)).

### 3.4. Mitochondrial Function Was Restored by Liquiritigenin

After long-term CCl4 treatment, the transcript levels of PGC-1*α*, ND1, and Bcl-x of rat liver were reduced by 30 to 50% compared to control rats. In the liver of rats that received liquiritigenin on top of CCl4, the mRNA levels of PGC-1*α*, ND1, and Bcl-x were more than 30–50% higher than those of CCl4-treated rats (*P* < 0.05) (Figure 5(b)). The hepatic PGC-1*α*, ND1, and Bcl-x protein levels were also substantially reduced by chronic CCl4 exposure and the reduction was alleviated by liquiritigenin (Figure 5(c)).

## 4. Discussion

Liquiritigenin showed promising effects in treating chronic liver injuries and preventing liver cirrhosis and hepatocellular carcinoma. After 8 weeks of CCl4 exposure, the expression levels of PGC-1*α*, Bcl-x, and ND-1 were significantly decreased whereas those of NF-*κ*B and MMP-2 were markedly increased, which led to heightened oxidative stress, increased cell death, and destruction of liver structure. Treatment with liquiritigenin essentially prevented abovementioned pathological genes with better preserved liver histology, decreased ROS and MDA levels, muted cell death, and suppressed inflammatory and fibrotic responses.

**Figure 6:**
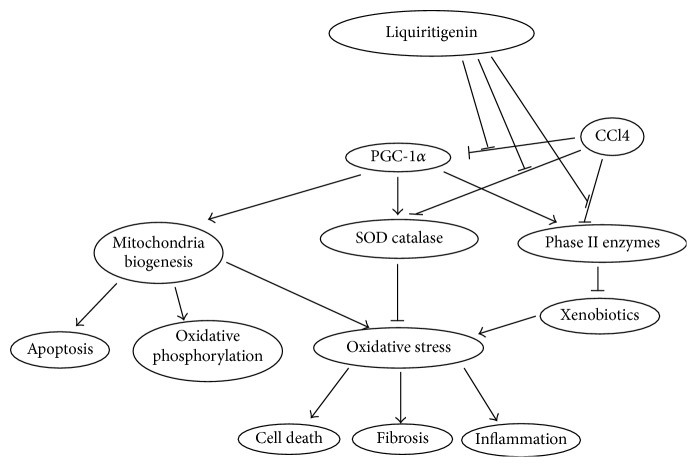
A proposed model on how liquiritigenin alleviates CCl4-induced liver injuries. CCl4 inhibits the expression of PGC-1*α*, indirectly inhibits the expression of genes involved in mitochondrial function, antioxidant, and phase II detoxification, and directly inhibits the activities of antioxidant enzymes and phase II enzymes, which leads to oxidative stress, cell death inflammation, and fibrosis. Liquiritigenin prevents or reverse CCl4-induced liver injuries by derepressing PGC-1*α* expression and relieving the inhibition of antioxidant enzyme and phase II enzyme activities, leading to normalized expression of genes involved in mitochondrial function, antioxidant, and phase II detoxification, reduced oxidative stress, cell death inflammation, and fibrosis.

Although the involvement of antioxidant and phage II enzymes is the common theme, many pathways have been implicated in the protective effects of various compounds against CCl4-induced liver injuries [8–10, 20–22]. Ursolic acid alleviated the inhibition of nuclear factor E2-related factor 2 (Nrf2) by CCl4 and restored the expression of Nrf2 downstream antioxidative genes to protect against liver fibrosis [20]. Nrf2 was shown to inhibit transforming growth factor- (TGF-) *β*/Smad signaling and plasminogen activator inhibitor- (PAI-) 1 expression, which led to inhibition of fibrosis [23]. Paraoxonases (PON) 1 and 3, which inhibit lipoprotein oxidation and atherosclerosis, and other antioxidant enzymes were involved in CCl4-induced liver injury in rats [21]. Ribosomal S-6 kinase acting downstream of mitogen-activated protein kinase (MAPK) pathway/extracellular signal-regulated kinase (ERK1/2) signal pathway to phosphorylate C/EBP-beta in hepatic stellate cells was shown to play a critical role in the progression of CCl4-induced liver fibrosis [24]. A variety of pathways have been shown to be involved in liver injuries leading to cirrhosis and hepatocellular carcinoma. As the master regulator of bioenergetic homeostasis and responses to oxidative stress, PGC-1*α* was shown to mediate the effects of liquiritigenin to relieve CCl4 induced chronic liver injury in rat (Figure 6).

PGC-1*α* is a transcriptional coactivator that was first identified as a regulator of mitochondrial biogenesis and function and regulates the genes involved in energy metabolism [25]. Ensuing studies have vastly expanded the functions of PGC-1*α*. PGC-1*α* (and PGC-1*β*) regulated macrophage polarization towards M2 and inhibited the expression of M1 proinflammatory cytokines to reduce inflammation during low-intensity exercise [26]. PGC-1*α* promoted physiological increase of mitochondrial content to protect L6 myoblasts against H_2_O_2_ induced apoptosis [26]. While promoting mitochondrial biogenesis, PGC-1*α* activated the expression of antioxidant enzymes including SOD2 and thioredoxin (Trx2) [27]. Moreover, activation of PGC-1*α* (and PGC-1*β*) mediated mitochondrial biogenesis was at the center of cytoprotective effects of many small molecules [28, 29]. Carbon monoxide/heme oxygenase-1 system worked through NF-E2-related factor-2/PGC-1*α*/nuclear respiratory factor 1 to induce mitochondrial biogenesis that led to prosurvival and anti-inflammatory effects during* Staphylococcus aureus* sepsis in mice [29]. It was clear that PGC-1*α* might play a central role in liver injuries leading to cirrhosis and liver cancers (Figure 6). CCl4 (and other insults) inhibited the expression of PGC-1*α*, which in turn repressed the expression of antioxidant enzymes including SOD2 and GSH-Px, mitochondrially encoded oxidative phosphorylation genes including ND1, and mitochondrial antiapoptosis genes including Bcl-x. The resulting oxidative stress caused inflammation, cell necrosis, and fibrosis. Without effective intervention, these pathological changes would lead to the end stage diseases liver cirrhosis and/or hepatocellular carcinomas. Liquiritigenin effectively blocked the pathological effects of CCl4 starting right from derepression of PGC-1*α* expression, which recovered the expression levels of antioxidant enzymes, mitochondrial function regulators, inflammatory and fibrogenetic genes, and apoptosis regulators. These changes led to reduced oxidative stress, cell death, and liver structure destruction during chronic CCl4 exposure.

In conclusion, liquiritigenin showed strong efficacy in inhibiting chronic exposure of CCl4 caused hepatic injuries through PGC-1*α* mediated derepression of antioxidant enzymes, oxidative metabolism genes, and mitochondrial pathway antiapoptotic genes. Moreover, it also inhibited inflammatory and fibrogenetic responses in rat liver upon CCl4 exposure.
